# The Journal of Comparative Medicine and Veterinary Archives for 1893

**Published:** 1893-01

**Authors:** 


### EDITORIAL.

We have to thank our readers for their appreciation of the value of the JOURNAL OF COMPARATIVE MEDICINE AND VETERINARY ARCHIVES as evidenced by our increasing number of subscribers, and by the many complimentary personal letters which have accompanied the renewal of subscriptions for the coming year. Under the name of the ARCHIVES OF COMPARATIVE MEDICINE AND SURGERY the JOURNAL was established in 1881 as a quarterly, proposing at the time to collate its matter, to a large degree, from foreign journals. The following year the name was changed to the JOURNAL OF COMPARATIVE MEDICINE AND SURGERY, as it was found that an active interest had been awakened in the public, and that the periodical was not to be a mere repository of already published matter, as indicated by the name ARCHIVES, but that the liberal contributions of original matter entitled it to the name of journal. For ten years we continued as a quarterly, but we then found that with the increase of veterinary societies and new discoveries, our readers could not wait two or three months for the reports of the proceedings of the veterinary world, and we commenced monthly publications, adopting our present dual title as more expressive of the scope and character of our work.

The material at our disposal has so increased, that each year we find the JOURNAL containing more pages, and some monthly numbers almost reaching the size of a former quarterly issue. For Volume XIV., 1893, which this number commences, we have greater hopes than we have had in any of our past progeny. We are promised a number of valuable, original articles, several of them from the pens of scientists who have neglected veterinary literature for some time. We have made arrangements for a methodical office supervision of the publication, which will avoid the delays in issue and errors of typography, which have, in the last year, been unavoidable on account of the personal business demands upon the time of the Editors.

In addition to the scientific articles already promised, we will have several of important practical interest, and we will do our utmost to report promptly the proceedings of societies and the changes and items of interest from the veterinary colleges.

We have several favors to ask of our contributors :

I. Send all matter to us promptly, at once, when completed, while it is still NEWS.

II. State if the papers are ORIGINAL, or if they have been previously published, and if copies have been sent to other papers for publication.

III. *Do not abbreviate.*

*Give titles in full.*

*Be accurate in dates, names and titles of persons and places.*

We are glad to make ordinary editorial corrections in manuscript, but we do not care to assume the responsibility of making extensive alterations where the sense of the matter is not perfectly clear.

When we assumed the business management of the JOURNAL two years ago we found the books in the obsolete condition in which those of many medical journals are still kept. Subscribers had been carried for years without payment of subscription. Out of courtesy to our readers, many of whom we recognized as only careless in these matters, we hesitated to make a radical change at once. Last year we commenced reasonable business methods with all new subscribers; we will now extend them to all.

We propose to furnish a live, interesting veterinary journal. To do this we must pay for material, illustrations, and for our printer's work. We do not wish to annoy our readers with constant bills, or to waste postage, or waste journals on our subscribers' widows, who do not think of ordering the JOURNAL discontinued.

From this issue all subscriptions must be paid in ADVANCE.

$3.00 a Year.

0.30 a Single Copy.

We beg that all delinquent subscriptions shall be paid before January 20th, 1893, as on that date we wish to use our new subscription books.

